# A solution to minimum sample size for regressions

**DOI:** 10.1371/journal.pone.0229345

**Published:** 2020-02-21

**Authors:** David G. Jenkins, Pedro F. Quintana-Ascencio

**Affiliations:** Department of Biology, University of Central Florida, Orlando, Florida, United States of America; Texas A&M University, UNITED STATES

## Abstract

Regressions and meta-regressions are widely used to estimate patterns and effect sizes in various disciplines. However, many biological and medical analyses use relatively low sample size (*N*), contributing to concerns on reproducibility. What is the minimum *N* to identify the most plausible data pattern using regressions? Statistical power analysis is often used to answer that question, but it has its own problems and logically should follow model selection to first identify the most plausible model. Here we make null, simple linear and quadratic data with different variances and effect sizes. We then sample and use information theoretic model selection to evaluate minimum *N* for regression models. We also evaluate the use of coefficient of determination (R^2^) for this purpose; it is widely used but not recommended. With very low variance, both false positives and false negatives occurred at *N* < 8, but data shape was always clearly identified at *N* ≥ 8. With high variance, accurate inference was stable at *N* ≥ 25. Those outcomes were consistent at different effect sizes. Akaike Information Criterion weights (AICc *w*_*i*_) were essential to clearly identify patterns (e.g., simple linear vs. null); R^2^ or adjusted R^2^ values were not useful. We conclude that a minimum *N* = 8 is informative given very little variance, but minimum *N* ≥ 25 is required for more variance. Alternative models are better compared using information theory indices such as AIC but not R^2^ or adjusted R^2^. Insufficient *N* and R^2^-based model selection apparently contribute to confusion and low reproducibility in various disciplines. To avoid those problems, we recommend that research based on regressions or meta-regressions use *N* ≥ 25.

## Introduction

*Limbo (noun)*: *(1) A place or state of neglect, oblivion, or uncertainty; (2) A dance or contest that involves bending over backwards to pass under a low horizontal bar*

All researchers seek to avoid their work being cast into the first definition of limbo, often by increasing sample size (*N*) and by applying increasingly sophisticated analytical techniques. But more samples require more effort, cost, and bodily risk (e.g., in field research). Researchers should then also find how low they can go in *N*, as in the second limbo definition above. To bend over backwards in that limbo dance is difficult, as is the process to clearly identify a minimum *N* needed for a study. In an era of big data, this may seem to be a former problem. In fact, it remains vital because multiple disciplines use data that are hard to acquire and/or aggregated. For example, it is difficult to collect data on species diversity among multiple islands with different areas. A similar problem occurs where data are aggregated, as in meta-analyses, systematic or quantitative reviews, and meta-regressions to evaluate general patterns across multiple studies (e.g., [1,2,3,4]). Consider a meta-analysis of 15 observational studies on a link between diet and cancer risk. Analyses may represent tens of thousands of surveyed individuals, but *N* = 15 for meta-analysis of the aggregated data. A regression computed with those aggregated data is called a meta-regression, and bears the same fundamental principles and assumptions as for a regression of the island diversity data.

Advanced regression methods may also apply to both scenarios. Similar to mixed-effects regressions that represent fixed and random effects, recent meta-regression methods can include proxies for variation among individuals as random effects in mixed-effects models, where the example *N* = 15 (above) represents the fixed effects [5,6,7]. Mixed-effects models likely require more *N* to characterize random effects than simpler models evaluated here. We return to mixed-effects models below, but results obtained here should set a lower limit for regressions and meta-regressions alike.

**Fig 1:**
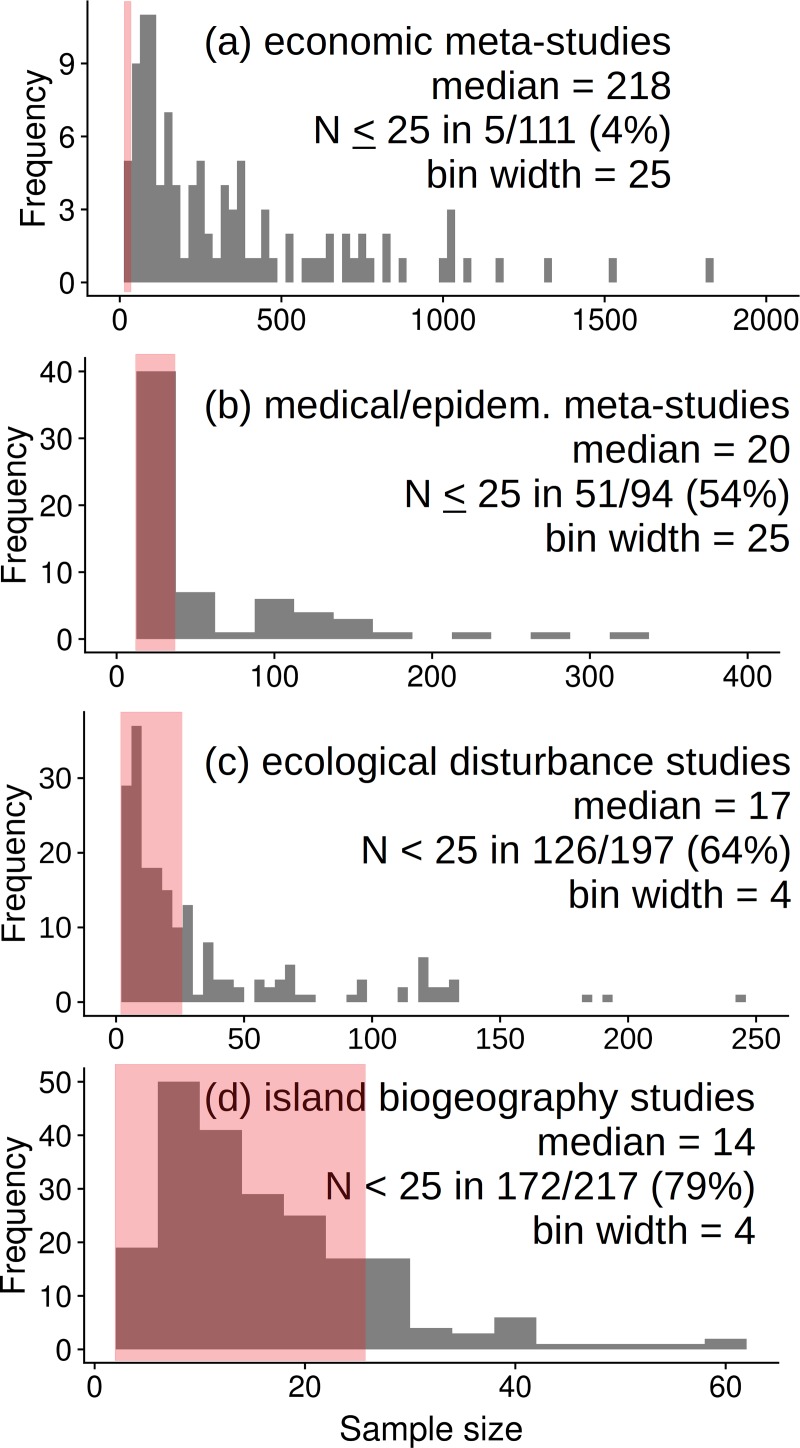
Histograms of *N* in research. (a) economic meta-analyses & meta-regressions; (b) medical / epidemiological meta-analyses & meta-regressions; (c) ecological analyses of disturbance [8]; and (d) biogeographical analyses of species-area relationships [9]. Please see S1 Appendix for a description of literature search methods, data, and references for (a) and (b).

Sample sizes tend to be relatively small in biological and medical disciplines (Fig 1). For example, economics tends to use hundreds of samples in meta-analyses and meta-regressions (median = 218; Fig 1A), but most medical and epidemiological meta-analyses tend to have far fewer samples (median = 20; Fig 1B; see S1 Appendix for a summary of search methods, results, and sources of those values). Sample size is even more limited where samples are difficult to obtain and data are then aggregated before analysis (e.g., the island species richness example, above). For example, nearly two-thirds (64%) of ecological disturbance studies [8] had N < 25 (median = 17; Fig 1C), as did nearly 4 of 5 (79%) studies of species-area relationships [9] in biogeography (median = 14; Fig 1D). A potential, general relationship between research funding and sample size among disciplines may exist, but the important point here is that diverse biological and medical research apparently use relatively small *N*.

The problem with small *N* is that inconclusive or contradictory results are more likely, especially given substantial variation [10,11,12]. This problem is well known; total citations for those three papers = 11,268 (Google Scholar, 2 September 2019). However, this problem persists (Fig 1), and so it is useful to consider this conundrum before exploring a solution. Small *N* has been discussed as one part of a larger reproducibility problem, where recommended solutions include registered studies, conflict of interest statements, and publication of data [11,13,14,15,16,17]. Greater sample size is often suggested (e.g, [12]) but a quantitative minimum *N* is rarely recommended. At least one journal now requires a minimum *N* = 5 per group for statistical analyses [18]. Ecological studies have been advised to use *N* = 10–20 per predictor [19] or *N* = 30–45 if studying gradients [20]. Others have offered advice based on the number of predictors (*p*): *N* > 50 + *p* [21]; *N* ~ 50 * *p* [22], or *N* > 50 + 8 * *p* [23]. Suggested minimum *N* clearly varies, if a value is provided.

It has been difficult to obtain consistent, clear guidelines for minimum *N* because that work has been based for decades on statistical power, which is the chance that a null hypothesis can be correctly rejected [24]. Power analysis is awkward for fundamental and operational reasons. Power analyses carry forward fundamental problems with null hypothesis inference, which is the long-standing basis for statistical analyses but that has been recently and widely criticized for several reasons [25,26,27,28,29]. Briefly, it assumes that the null hypothesis is meaningful, whereas research is typically conducted on the premise that an alternative model may be supported. Thus, statistical tests and research are usually mismatched in assumptions, and the underlying logic is convoluted when evaluating statistical output [26,27]. Also, the arbitrary p ≤ 0.05 criterion for statistical "significance" and its numerous work-arounds have been widely discussed, including *post hoc* hypothesis formation, data dredging, and p-hacking [30,31,32]. Finally, null hypothesis testing is now recognized as relatively weak inference compared to other approaches [33]. Instead, inference is stronger when based on comparison of multiple models representing alternative hypotheses, including a null [26].

The use of power analysis to estimate minimum *N* also suffers from a fundamental cart-before-the-horse problem. Consider an experiment to evaluate three alternative hypotheses that predict either a sloped linear model, a humped-shape curve, or null data pattern. A typical and straightforward power analysis for regressions (e.g., pwr.f2.test in the R pwr package [34]) applies only to the linear model–before finding which shape best represents the data. In principle, a power test is possible for a hump-shaped model [35], but conventional statistical power tests do not include that possibility. This fact runs counter to strong inference based on multiple working hypotheses [33,36,37,38] because only one of the hypotheses can be evaluated for statistical power. Studies designed with this approach may not be able to fully evaluate the hump-shaped prediction.

Operationally, power analysis is a challenging way to estimate minimum *N* because there are four interacting parts. A researcher solves for *N* by assuming the remaining three: a desired power level (typically ≥ 0.80); effect size (i.e., slope in linear regressions, or elasticity in economics); and significance level (typically p = 0.05) [11,24,39,40,41,42]. Preliminary data can help those assumptions but are not always available or predictive. A consequent challenge emerges because an expected effect size becomes a goal of the research. But if an effect size is expected so well that subsequent research is based on it, then a Bayesian, confirmatory analysis is more appropriate than a frequentist, null hypothesis inference framework that uses statistical power [43]. Bayesian approaches analogous to power exist [44,45] but have not yet been widely applied to this problem.

A separate operational problem arises because alternative models are often selected using a coefficient of determination (R^2^) or the adjusted R^2^ that accounts for differences in model complexity. That practice is ill-suited to select among alternative models, especially if models differ in the number of parameters and if regression assumptions are violated [38,42,46]. Instead, model selection is now preferred to be based on information theory metrics and parsimony [26,38], according to the logic of Occam’s razor (“shave away all but what is necessary”). Adjusted R^2^ can then be used to “criticize” the fit of a selected model [46], essentially applying Whitehead’s caveat to Occam’s razor: “seek simplicity but distrust it” [47].

Fortunately, statistical advances using information theory enable a different approach [26,38] that resolves the above problems. Here we use that approach to identify a minimum *N* needed to clearly identify the shape of data made with null, simple linear, and quadratic regressions. We simulate data across a range of variances and effect sizes, and then solve regression models at a range of *N* to find a minimum N where the data match the regression model. Our approach is purposefully simple to help make it approachable, but we hope the above background and Fig 1 demonstrate that the subject is far from trivial.

This work has boundaries. Between the limits of a perfectly fitted model (where every point is on a line (R^2^ = 1.0) and random scatter (R^2^ = 0) there lies a practically infinite set of combinations for the factors affecting power of regressions (i.e., variance * effect size * *N)*. We concentrate on four corners of a variance X effect size grid, where the four choices represent low & high combinations of effect size and variance. Having established those approximate margins for a data shape (e.g., straight-line pattern), we repeatedly evaluate regressions with different *N*.

We restrict work here to 1^st^ and 2^nd^-order polynomial linear models, which are two members of the *class* of linear models, so named because they include additive combinations of constants and coefficients multiplying a predictor variable (x). Within that class, the 1^st^-order or simple linear model (y = *α* + *β*x + ε) is often dubbed the linear model. To avoid confusion between the class and its models, we hereafter refer to the “straight-line” model in the linear class. A 2^nd^-order polynomial is also a linear model and often dubbed the quadratic equation (y = *α* + *β*x + *γ*x^2^ + *ε*), which is the most parsimonious first step to evaluate curvature beyond a straight-line model [48].

We set aside here multiple regressions (i.e., including covariates) but results should apply (discussed below). We also do not include higher-order polynomials because we know of no major hypotheses that predict them. Instead, fitting higher-order polynomials seems to be more often used in *post hoc* trend-fitting (e.g., temporal patterns). We also set aside nonlinear models for two reasons. Curved data are often transformed to fit straight-line models (e.g., [9,49,50]), so much evidence on important curvilinear ideas is actually based on straight-line models. Also, nonlinear models are sensitive to required initial parameter values and thus difficult to solve (contributing to the first reason). Future work may extend the approach here to nonlinear models.

Finally, we use the Akaike Information Criterion (AIC) to select the most plausible model among the analyzed set. A conceptual continuum exists between hypothesis refutation and confirmation, where a Bayesian version (BIC) is targeted to confirm a true hypothesis (e.g., a particular model with expected coefficients), and AIC is aimed at exploratory model selection in a frequentist context [43]. The BIC might seem appropriate at first glance because we evaluate predefined models. However, much empirical research conducted by others does not share the luxury of already knowing a “true” pattern. Instead it evaluates alternative hypotheses by exploring observed patterns and solving for the most predictive model coefficients. To be most useful to research by others, we make data and then use AICs to identify the most likely model in a set. Results were evaluated for the following specific questions and expectations:

1. What is the minimum *N* needed for accurate inference (i.e., the match of alternative models to data classes)? We expected that insufficient *N* would interfere with accurate inference (including false positives and false negatives), but that some threshold *N* may exist, where accurate and consistent inference is always reached). This was best evaluated for data sets with low variance.
2. How does variance alter the answer to question 1? We expected that general patterns from above would hold true, but that minimum *N* would be increased by variance.
3. How does the use of AICc *w*_*i*_ vs. adjusted R^2^ alter interpretations above? Based on statistical texts cited above, we expected adjusted R^2^ values to less accurately identify the correct data class than AICc *w*_*i*_ values. This was evaluated by comparing AICc *w*_*i*_ and adjusted R^2^ values in plots.

## Materials and methods

Three classes of data (i.e., random (null), straight-line, and quadratic) defined above were generated with N = 50, simply by prescribing a model and then adding variance. Thus, data sets simply represented scatter plots with little or much variation added. We chose N = 50 to exceed most data sets of interest here (Fig 1B–1D). Model parameters were aimed to extremes of variance and effect size (as in four corners of a variance X effect size grid), with the goal that high variance made it difficult to detect the true pattern (e.g., visually and as indicated by a low adjusted R^2^ and a weakly significant coefficient). Alternatively, low variance made an obvious pattern closely adhering to a model. Because these extremes are approximate, we treated outcomes as approximate and made cautious recommendations. We anticipated that data sets with high variance would be most interesting because they most resemble empirical data collected in complex scenarios.

Two null (random; slope = 0) data sets were created to represent low and high variance in the intercept term (*α*). Four straight-line data sets were created with low or high slopes (*β*) and low or high variance in *β*. Eight quadratic data sets were created because the second coefficient (*γ*) was added to the straight-line process and also evaluated for its own effect size X variance combinations. In total, 14 data sets then evaluated for each of null, straight-line, and quadratic models. Coefficients and variance (modeled as standard deviation of residuals, σ) used to make data sets are listed in S1 Table. Generated data are shown in Results below.

Analyses were conducted as follows (see R code in S2 Appendix). A sample of *N* = 4 was taken from a full data set (*N* = 50). That minimum *N* = 4 was set by the minimum degrees of freedom for a quadratic model because all comparisons included the null, straight-line, and quadratic models. The sample was evaluated for each of the 3 models, and models were compared by weights (*w*_*i*_) for corrected AIC (AICc) values. A *w*_*i*_ value is the preferred criterion for model selection because it scales from 0–1 to indicate the probability that a model is most plausible. Corrected AIC values adjust for smaller *N*, and approach uncorrected AIC values at *N* ~ 40 [38]. The *w*_*i*_ value and an adjusted R^2^ value for each model was recorded. That process was repeated 99 more times at that *N* (i.e., sampling with replacement from the initial data), so that mean *w*_*i*_ and adjusted R^2^ values (with 95% confidence intervals) could be computed for the 100 replicates at that *N*. That whole process was then repeated from *N* = 5 to *N* = 50 for a total of 4,700 AIC comparisons per data set (197,400 AIC comparisons overall). Mean *w*_*i*_ and adjusted R^2^ values (with 95% confidence intervals) were plotted as functions of *N* for each combination of a data set and model. In addition, approximate *N* where *w*_*i*_ values for one model surpass those of another model were evaluated graphically.

## Results

To reiterate findings above, most meta-analyses and meta-regressions in medicine and epidemiology have much smaller *N* than similar analyses in economics (Fig 1). Studies of ecological disturbance and species-area relationships in biogeography tend to have even lesser *N*. Results below should be important to multiple biologically-based disciplines.

**Fig 2:**
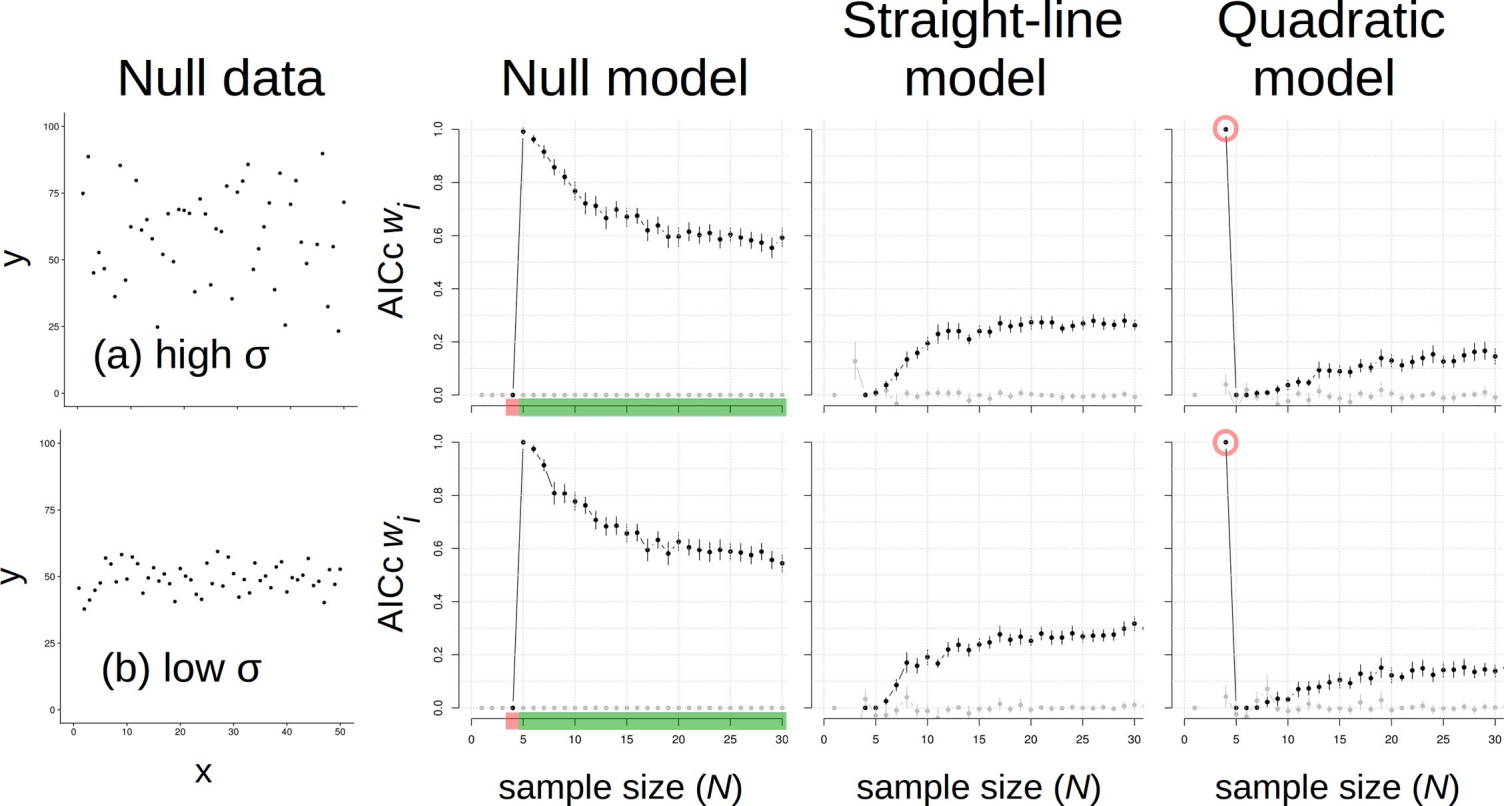
Data made with a null model (1^st^ column) and results of analyses using null (2^nd^ column), straight-line (3^rd^ column) and quadratic (4^th^ column) models. Data with (a) high variance and (b) low variance were each analyzed at *N* = 4–50. Results are presented with maximum *N* = 30 for visual clarity; all results stabilized at *N* > 30. Circles are means; error bars are 95% confidence intervals. “Traffic signal” colors on sample size (*N*) axes for the null model indicate ranges where *N* is too small (red = stop), or sufficient (green = go) to correctly infer the pattern. Note the quadratic model outcomes at *N* = 4 (red circles).

Analyses of null data represented an extreme edge of the conceptual variance X effect size grid because there was no effect size (i.e., slope). Interestingly, a null model was implausible (i.e., mean AICc *w*_*i*_ = 0.0 for 100 replicates) for null data with *N* = 4 because a quadratic model was always most plausible, regardless of variance in the data (*w*_*i*_ = 1.0; Fig 2). Essentially, a plausible curved line can always be drawn for 4 data points, and this pattern was consistent at both low and high σ. But adding one more datum reversed that outcome, so that the null model was now always most plausible with *N* = 5 and the quadratic was always less plausible. The null model remained most plausible with greater *N* (Fig 2), though *w*_*i*_ values declined progressively. A straight-line model was more plausible than the quadratic at *N* ≥ 7 but never exceeded null model values of *w*_*i*_ (Fig 2). We interpreted these results to indicate that *N* ≤ 7 should not be used to compare quadratic to straight-line and null models, even if patterns are tight around lines.

**Fig 3:**
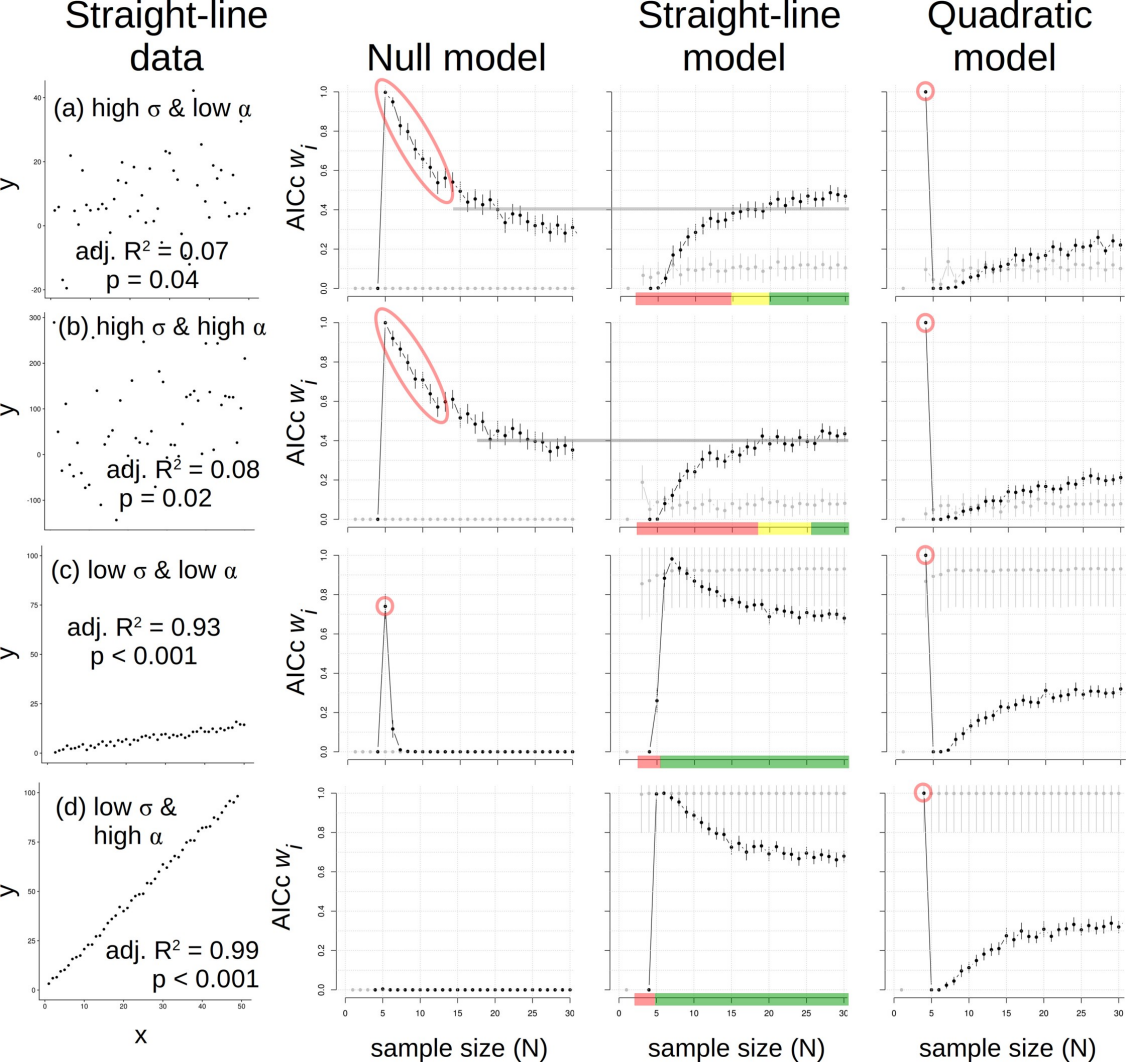
Data made with a straight-line model (1^st^ column) and results of analyses using null (2^nd^ column), straight-line (3^rd^ column) and quadratic (4^th^ column) models. The four combinations (a-d) of low/high variance (*σ*) and effect size (*α*) represent approximate graphical extremes. Grey lines represent transitions in leading *w*_*i*_ between two models. “Traffic signal” colors on sample size (N) axes for the straight-line model indicate ranges where *N* is too small (red = stop), about equivalent to the null (yellow = caution), or sufficient (green = go) to correctly infer the pattern. Note the null and quadratic model outcomes at low *N* (red circles or ellipses).

Data generated with a straight-line model represented all four combinations of low and high σ X effect size (Fig 3). The switch between quadratic and null models at N = 4 & 5 occurred again in every case for straight-line data, regardless of σ or slope (Fig 3). With high variation and a low slope, the *w*_*i*_ for the null model decays slowly; evidence that the straight-line model is most plausible finally exceeds evidence for the null at *N* > 20 (Fig 3A). A similar outcome was observed for straight-line data with high variation but a greater slope (Fig 3B), though the transition in *w*_*i*_ values occurred at *N* ~ 25. In both cases with high σ, we concluded that *N* ≥ 25 would be most able to accurately detect a straight-line pattern using AICc *w*_*i*_ values. In contrast, analyses of high variance patterns with fewer *N* will incorrectly support an inference of a quadratic (*N* = 4) or null (*N* = 5 to ~ 25) pattern. Adjusted R^2^ values for straight-line and quadratic models were similar at all *N* > 5 and would not help identify the matching model (Fig 3A & 3B).

Model fits to straight-line data with relatively low σ and low slope (Fig 3C) more simply echoed the patterns above: the quadratic model was most plausible at *N* = 4, the null was most plausible at *N* = 5, but thereafter the straight-line model was most plausible. That general pattern was repeated for straight-line data with low σ but relatively high slope (Fig 3D), except the low σ and high slope combination prevented the null from being most plausible at *N* = 5. Straight-line models for tightly straight-line data maintained highest *w*_*i*_ values beyond *N* = 5 with slight decay. Again, adjusted R^2^ values could not distinguish between straight-line and quadratic models at all *N* (Fig 3C & 3D).

**Fig 4:**
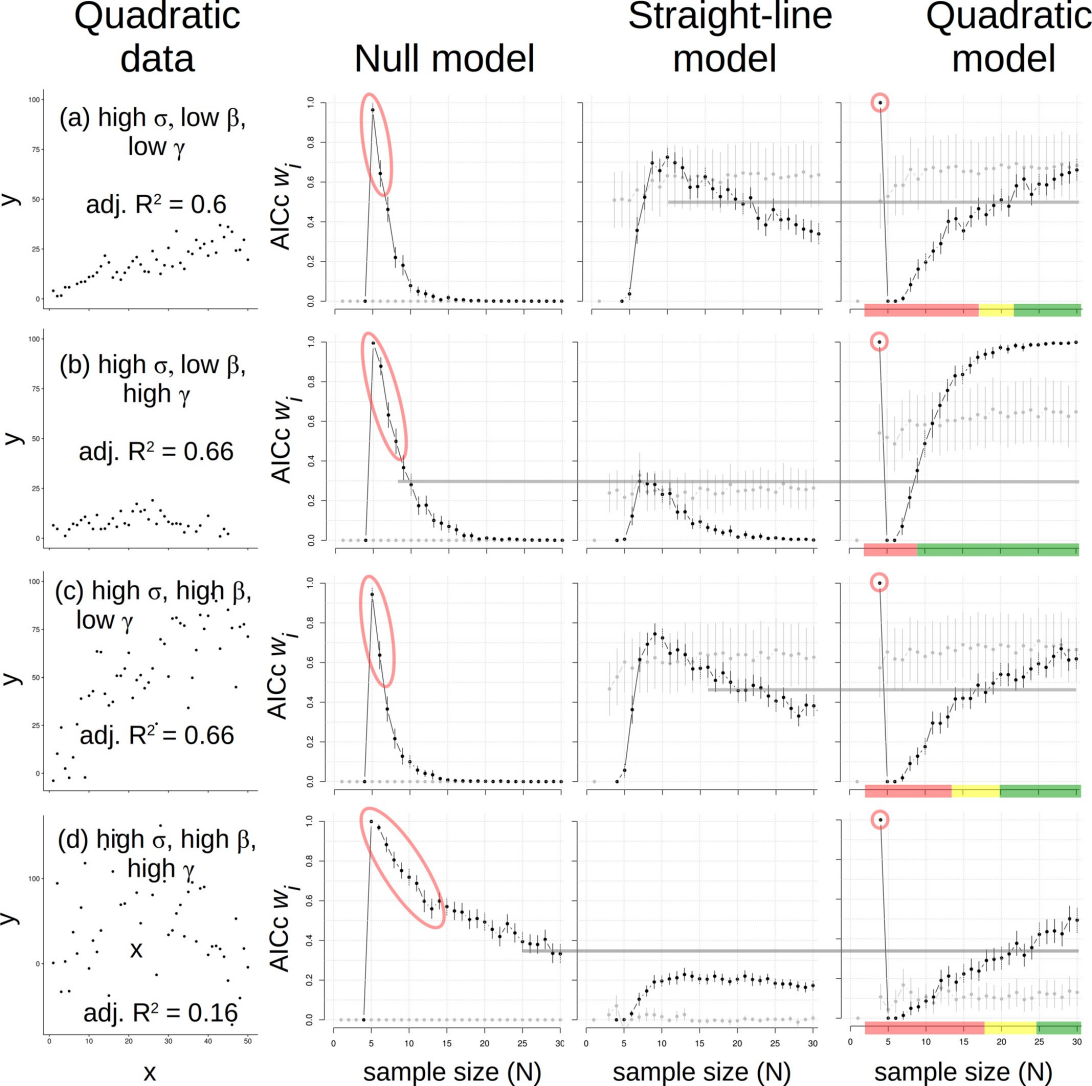
Data made with a quadratic model and with high variance (*σ;* 1^st^ column) and results of analyses using null (2^nd^ column), straight-line (3^rd^ column) and quadratic (4^th^ column) models. All else as in Figs 2 & 3.

**Fig 5:**
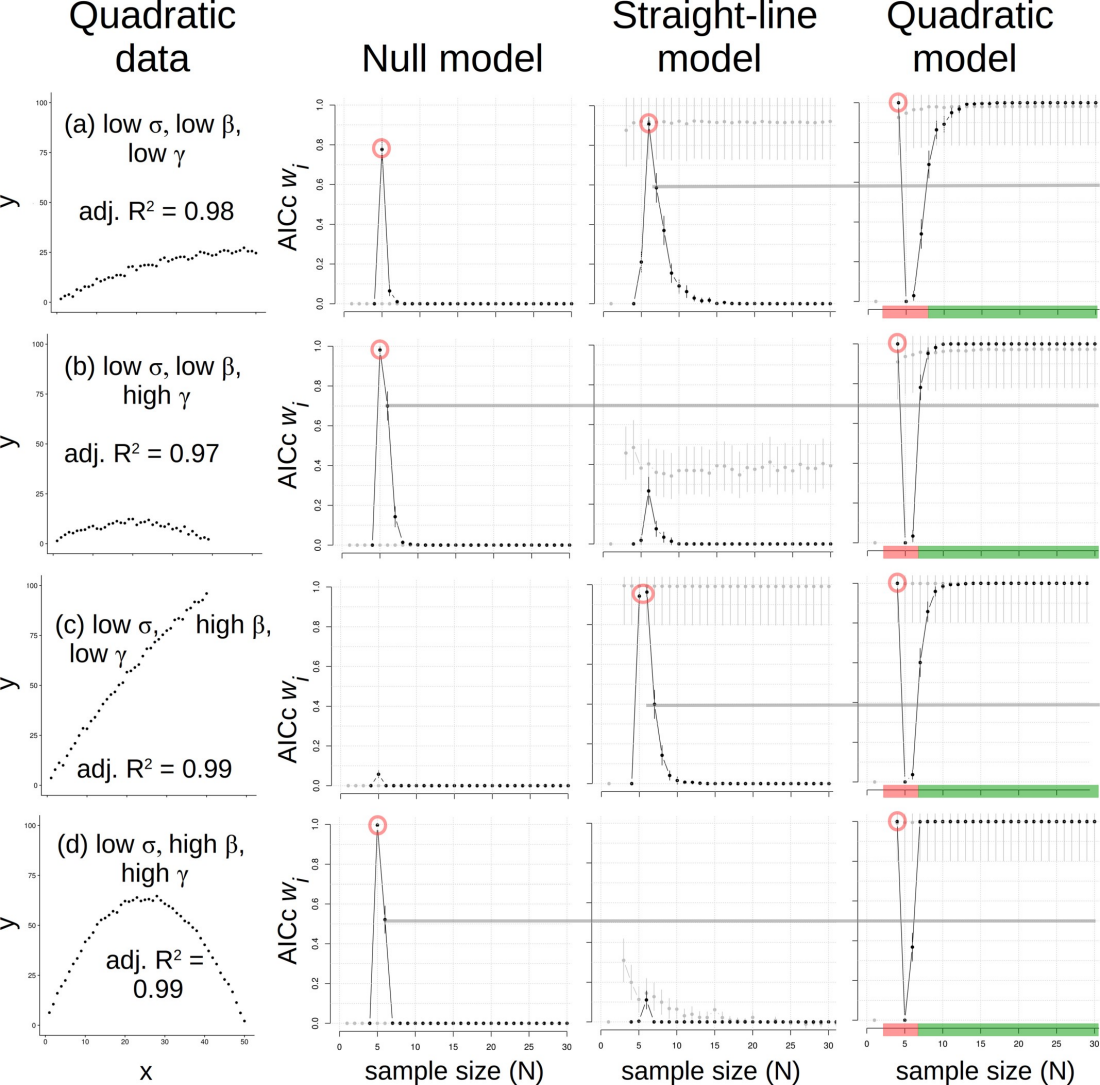
Data made with a quadratic model and with low variance (*σ;* 1^st^ column) and results of analyses using null (2^nd^ column), straight-line (3^rd^ column) and quadratic (4^th^ column) models. All else as in Figs 2–4.

Data generated as a quadratic function had low and high effect size for two coefficients (*β*, *γ*) and low and high σ, so the σ X effect size grid was effectively a cube. We organized results for high σ outcomes (Fig 4) and then low σ outcomes (Fig 5). For high σ results, the same pattern at *N* = 4–5 was repeated; first the quadratic, then the null model was most plausible (Fig 4). At *N* > 5, the null became progressively less plausible when the quadratic data appeared roughly linear (i.e., had low *γ*; Fig 4A & 4C), and the straight-line model was most plausible at intermediate *N* (i.e., 5 < *N* < 20). In those cases, the quadratic model most plausibly represented quadratic data only at *N* > 20 (Fig 4A & 4C). Where greater *γ* was used to make data pattern appear more curved (Fig 4B & 4D), the quadratic model became most plausible at N ≈ 8 given a tight pattern (Fig 4B) but at N ≈ 25 given a scattered pattern (Fig 4D). Thus, accurate inference of a quadratic model depended greatly on *N*, σ, and effect size, where either the null or the straight-line could inappropriately appear most plausible at insufficient *N*. We concluded that *N* ≥ 25 is needed to correctly detect a quadratic pattern using AICc *w*_*i*_ values with relatively high σ in the data (which should be expected *a priori* if a researcher is cautious). Adjusted R^2^ values only helped to identify the quadratic when *γ* was high, which makes sense because that term is what differs between straight-line and quadratic models. In other words, given a weak quadratic effect (low *γ*), adjusted R^2^ could not accurately identify the data shape, though AIC *w*_*i*_ values could (given sufficient *N*).

When quadratic data were made with low σ (Fig 5), general patterns for the variable quadratic analyses (Fig 4) were repeated, but with sharper boundaries between models. As before, the quadratic and null models traded places as most plausible at *N* = 4 and 5, respectively. But now with low σ, the null model did not linger at greater *N* as plausible (Fig 5). Likewise, the straight-line model repeatedly peaked in *w*_*i*_ values at *N* = 6 (essentially in the “valley” of quadratic *w*i values; Fig 5). Quadratic models repeatedly regained primacy at *N* ~ 8 and remained so.

## Discussion

Low sample size contributes to problems of reproducibility, including false positives and false negatives and apparently contributes to uncertainty in biology and medical sciences [8,9,11,12,14,16]. Most attention on sample size has focused on power and effect size, as well as matters of study design and biases [11,12]. We approached the matter of sample size differently by addressing a question that should be answered before evaluating effect sizes: What is the minimum N needed to correctly match a model to a data shape? That question is handled by model selection, where models should represent alternative hypotheses [26,38].

The answer depends on variance, but importantly, not on effect size or the model (straight-line or quadratic). Where one must evaluate support for alternative hypotheses predicting null, straight-line, or quadratic regression models, we recommend a minimum *N* = 8 for a tight data pattern (i.e., very low variance). But with high variance, minimum *N* is pushed to *N* ≈ 25 to clearly match a model to the data pattern. That answer represents the upper edge of the variance X effect size grid analyzed here, and represents a cautious recommendation for many observational studies that rely on regressions, including meta-regressions. The expectation that effect size would alter the answer was inherited from power analyses, which focus on statistical significance of a slope coefficient. That did not translate to the AIC-based model selection used here, where the answer did not depend on statistical significance.

We also compared AICc *w*_*i*_ to adjusted R^2^ for the interpretations above. Results here confirm existing recommendations that R^2^-based values do not clearly identify the data shape [38,42,46] though it continues to be widely used for that purpose. This practice needs to be abandoned, and our collective understanding of past research built on comparisons of R^2^ values among alternative models needs to be re-evaluated. Going forward, we echo Bolker’s [46] recommendation that researchers first compare models using AIC (or BIC), and then use R^2^ or adjusted R^2^ to “criticize” goodness-of-fit for the most plausible model.

The main recommendations above (*N* ≥ 8 with very little variance, but *N* ≥ 25 with any more variance) assume samples are not clustered at one end of a data cloud, and regression assumptions are met. Models here did not include covariates, which add a degree of freedom per covariate but can help “explain” variation in empirical data and yield better coefficient estimates. Relatively weak (i.e., scattered) evidence for ideas at low *N* might be “rescued” with important covariates, especially if predictors are scaled to standardize varying units. For example, a regression to predict risk of a disease as a function of body weight should include important covariates (demographics, health history, etc.), which may affect disease risk more than body weight. Careful planning and foreknowledge of the study system may help ensure that the most fruitful covariates are measured [42]. Also, analyses here used only fixed effects, but can inform mixed-effects regressions and meta-regressions increasingly used in natural sciences and medical research. Estimating sample size for mixed-effects models is complicated because it depends on having enough random factor levels and samples within those levels to characterize random variation. In addition, correlation between random levels is important [51]. Thus, our recommended minimum N ≥ 25 for fixed effect models is surely too low for many mixed-effects models. Therefore, mixed-effects regressions and meta-regressions with random effects [6,52] are very likely to require *N* >> 25 to adequately represent data patterns. These considerations emphasize that skepticism should be applied to mixed-effects regressions and meta-regressions with *N* ≤ 25 and without well-described variance and correlation structures.

In summary, statistical limbo may be better avoided and reproducibility improved if research based on regressions and meta-regressions uses *N* ≥ 25. This cautious recommendation is based on analyses that use information theory rather than power analyses encumbered by fundamental and operational problems. Greater N is likely needed for regression models more advanced than those used here. Results here bear important implications for the way future research is conducted and how past research is interpreted for some important subjects in biology and associated professions.

For example, results here indicate that insufficient N has been used in ~1/2 of medical and epidemiological meta-analyses and meta-regressions, ~2/3 of ecological disturbance studies, and ~4/5 studies of species-area relationships in biogeography. This fundamental problem contributes to uncertainty in subjects as disparate as benefits of exercise [53], linkage between binge drinking and heart disease [54], ecological disturbances [8,55,56,57] and the relationship between natural diversity and habitat area [9,58]. We expect other subjects share these problems, but re-analyses of past evidence using *N* ≥ 25 will better resolve uncertainties, and future research conducted with *N* ≥ 25 will better resolve patterns in regressions and meta-regressions.

## Supporting information

S1 Appendix Data for Fig 1(A) and 1(B) were obtained using Google Scholar searches on 24 July– 5 August 2019, based on the keywords listed in the figure caption and with a target number of papers per topic ~100, on the principle that a large sample size would adequately approximate medians and actual distributions. The only criterion for retention of a paper in data was that sample size (*N)* was listed. Data and citations for (a) and (b) are listed in medecontallies.xls.
(DOC)

S2 Appendix R code used for data generation and analyses. (DOCX)

S1 Table Coefficients and variance used to make data sets. Variance was set as standard deviation (*σ*) of residuals. Coefficients (*α*, *β*, *γ*) designate data shape, where *α* is the intercept, *β* is a multiplier for x (as in y = *α* + *β* x), and *γ* is a multiplier for the quadratic term (… + *γ* x^2^).
(DOCX)

S1 Data (XLS)
