# Supplementary material for: A solution to minimum sample size for regressions
Source: PLoS One. 2020 Feb 21;15(2):e0229345. doi: 10.1371/journal.pone.0229345 (PMC7034864; doi:10.1371/journal.pone.0229345)
Supplement: S1 Appendix — The only criterion for retention of a paper in data was that sample size (N) was listed. Data and citations for (a) and (b) are listed in medecontallies.xls. (DOC) [file pone.0229345.s001.doc]

**S1 Appendix.** **Data for Figs 1a & b.** Data were were obtained using Google Scholar searches on 24 July – 5 August 2019, based on the keywords listed in the Figure caption and with a target number of papers per topic ~100, on the principle that a large sample size would approximate medians and actual distributions adequately. The only criterion for retention of a paper in data was that sample size (*N)* was listed. Data and citations for (a) and (b) are listed in medecontallies.xls.
