# Supplementary material for: A solution to minimum sample size for regressions
Source: PLoS One. 2020 Feb 21;15(2):e0229345. doi: 10.1371/journal.pone.0229345 (PMC7034864; doi:10.1371/journal.pone.0229345)
Supplement: S2 Appendix — (DOCX) [file pone.0229345.s002.docx]

**S3 Appendix. R code used for data generation and analyses.**

# R code to test for the effect of different sample sizes on AICc weights of linear regression models

# Pedro Quintana-Ascencio with edits by Dave Jenkins, 21 June 2019

require(ggplot2)

require(cowplot)

require(bbmle)

rm(list=ls()) #will remove ALL objects

gc()# garbage collection to remove unneeded junk from memory

N= 50 # maximum number of samples possible

s <- seq(1,50,1) # number of Sampled data (from N) - to be graphed below

Iterations <- 100 # number of iterations

out1<-out2<-out3<-out4<-out5<-out6 <- array(0,c(length(s),Iterations)) # make empty arrays to fill below

table_aics <- NULL # empty table to hold aic outcomes

##################### Step 1 = make data. Here null, straight, or quadratic

##### Do this in two ways: a. steep but scattered, and b. slight but consistent

##### Plots are important to visually verify shapes and variance

#### Make full set of Null Data

parms <- c(50,25) # set params for models; 50,20 gets decent scatter; 50,5 gets tight

x <- 1:N

y <- parms[1] + rnorm(length(x),0,parms[2])

tiff('your file location and name here.tiff', units="in", width=6, height=6, res=600)

qplot(x,y,ylim=c(0,100),xlim=c(0,max(x)), col=I("black"))

dev.off()

summary(lm(y~x))

#### OR Make Straight-Line Data

# 1,0.25,1 gets lo scatter / lo effect; 1,2,1 = lo/hi; 1,0.25,10 gets hi/lo; 1,2,100 gets hi / hi

parms <- c(1,2,1) #

x <- 1:N

y <- parms[1] + parms[2]*x + rnorm(length(x),0,parms[3])

tiff('your file location and name here.tiff', units="in", width=6, height=6, res=600)

qplot(x,y,ylim=c(0,100),xlim=c(min(x),max(x)), col=I("black"))

dev.off()

summary(lm(y~x))

#### Or Make Quadratic Data for each combo of b & c coeff's and sd below

# lo b, lo c, lo sd: parms <- c(1,1,-.01,1) low and slight but hily sig deflection R2 often ~ 0.98

# lo b, hi c, lo sd: parms <- c(1,1,-.025,1) low tight arch hily sig deflection R2 often ~ 0.97

# hi b, lo c, lo sd: parms <- c(1,3,-.015,1) hi and slight but hily sig deflection R2 often ~ 1.0

# hi b, hi c, lo sd: parms <- c(1,5,-.1,1) hi tight arch hily sig deflection R2 often ~ 1.0

# lo b, lo c, hi sd: parms <- c(1,1,-.01,5) low and slight sig p=0.004 deflection R2 = 0.6

# lo b, hi c, hi sd: parms <- c(1,1,-.025,4.5) low arch and hily sig deflection R2 = 0.66

# hi b, lo c, hi sd: parms <- c(1,3,-.015,10) hi and sig p=0.001 deflection R2 = 0.91

# hi b, hi c, hi sd: parms <- c(1,5,-.1,50) hi fuzzy arch sig p=0.0002 R2 = 0.16

parms <- c(1,5,-.1,50)

x <- 1:N

x2 <- x^2

y <- parms[1] + parms[2]*x + parms[3]*x2 + rnorm(length(x),0,parms[4])

tiff('your file location and name here.tiff', units="in", width=6, height=6, res=600)

qplot(x,y,ylim=c(min(y),max(y)),xlim=c(min(x),max(x)), col=I("black"))

dev.off()

summary(lm(y~x+x2))

######################## Step 2. Iteratively sample different N from data chosen abov e & run models

# run this first chunk; you will get a + in console because the for-loop is not yet done:

for (i in 1:length(s)) {

for (j in 1: Iterations){

x <- sample(1:N,s[i]) # w/o replacement

x2 <- x^2

# Now Choose the ONE model that matches the built data

y <- parms[1] + rnorm(length(x),0,parms[2]) # NULL

y <- parms[1] + parms[2]*x + rnorm(length(x),0,parms[3]) # STRAIGHT

y <- parms[1] + parms[2]*x + parms[3]*x2 +rnorm(length(x),0,parms[4]) # QUADRATIC

# Now run thru the #####s to make AICs table

anull <- lm(y ~ 1)

quad <- lm(y ~ x + x2)

straight <- lm(y ~ x)

table_aics <- AICctab(anull,straight,quad,base=T,delta=T,weights=T,sort=F, nobs=length(x))

out1[i,j] <- as.numeric(table_aics$weight)[1] # where [1] picks the anull model (Anull so it is always 1st alphabetically)

out2[i,j] <- as.numeric(table_aics$weight)[2] # where [2] picks the straight model (alphabetically) in the AIC table

out3[i,j] <- as.numeric(table_aics$weight)[3] # where [3] picks the quad model (alphabetically) in the AIC table

adjrn <- summary(anull)$adj.r.squared # extract and record adj R2 values from regressions

out4[i,j] <- as.numeric(adjrn)

adjrq <- summary(quad)$adj.r.squared # extract and record adj R2 values from regressions

out5[i,j] <- as.numeric(adjrq)

adjrs <- summary(straight)$adj.r.squared

out6[i,j] <- as.numeric(adjrs)

}}

table_aics # to see aic table for last iteration - anull, straight, then quad

########

# Now plot AIC weights ~ N

par(mfrow=c(1,1))

### plot NULL model AICc wi ~ sample size

r <- rowSums(out1)/Iterations

ar2 <- rep(0,length(s))

for (j in 1:length(s)) {ar2[j] <- mean(out4[j,])}

ar2ci <- 1.96*sqrt(abs(ar2)/Iterations)

# plot mean and 95%CI of adj R2 per sample size

tiff('your file location and name here.tiff', units="in", width=6, height=6, res=600)

plot(s,ar2,pch=19,cex=0.75,ylim=c(0,1),xlim=c(0,30),type='b',col='grey',ann=F,frame.plot=F)

for (i in 1:length(s)){

lines(c(s[i],s[i]),c(ar2[i]-ar2ci[i],ar2[i]+ar2ci[i]), col="grey")}

par(new=TRUE) # now overlay plot of weights ~ N

plot(s,r,pch=19,cex=0.75,ylim=c(0,1),xlim=c(0,30),type='b',col='black',ann=F,frame.plot=F)

v <- rep(0,length(s))

for (j in 1:length(s)) {v[j] <- var(out1[j,])}

v <- 1.96*sqrt(v/Iterations)

for (i in 1:length(s)){lines(c(s[i],s[i]),c(r[i]-v[i],r[i]+v[i]))} #

abline(v=c(seq(0,50,5)), col='lightgray', lty=3)

abline(h=c(seq(0,1,0.1)), col='lightgray', lty=3)

dev.off()

### plot STRAIGHT model AICc wi ~ sample size

r <- rowSums(out2)/Iterations

ar2 <- rep(0,length(s))

for (j in 1:length(s)) {ar2[j] <- mean(out6[j,])}

ar2ci <- 1.96*sqrt(abs(ar2)/Iterations)

# plot mean and 95%CI of adj R2 per sample size

tiff('your file location and name here.tiff', units="in", width=6, height=6, res=600)

plot(s,ar2,pch=19,cex=0.75,ylim=c(0,1),xlim=c(0,30),type='b',col='grey',ann=F,frame.plot=F)

for (i in 1:length(s)){

lines(c(s[i],s[i]),c(ar2[i]-ar2ci[i],ar2[i]+ar2ci[i]), col="grey")}

par(new=TRUE) # now overlay plot of weights ~ N

plot(s,r,pch=19,cex=0.75,ylim=c(0,1),xlim=c(0,30),type='b',col='black',ann=F,frame.plot=F)

axis(side=1, at=c(0,50,10,20,30,40))

axis(side=2, at=c(0,1,0.2,0.4,0.6,0.8))

v <- rep(0,length(s))

for (j in 1:length(s)) {v[j] <- var(out2[j,])}

v <- 1.96*sqrt(v/Iterations)

for (i in 1:length(s)){

lines(c(s[i],s[i]),c(r[i]-v[i],r[i]+v[i]))}

abline(v=c(seq(0,50,5)), col='lightgray', lty=3)

abline(h=c(seq(0,1,0.1)), col='lightgray', lty=3)

dev.off()

### plot QUAD model AICc wi ~ sample size

r <- rowSums(out3)/Iterations

ar2 <- rep(0,length(s))

for (j in 0:length(s)) {ar2[j] <- mean(out5[j,])}

ar2ci <- 1.96*sqrt(abs(ar2)/Iterations)

# plot mean and 95%CI of adj R2 per sample size

tiff('your file location and name here.tiff', units="in", width=6, height=6, res=600)

plot(s,ar2,pch=19,cex=0.75,ylim=c(0,1),xlim=c(0,30),type='b',col='grey',ann=F,frame.plot=F)

for (i in 1:length(s)){

lines(c(s[i],s[i]),c(ar2[i]-ar2ci[i],ar2[i]+ar2ci[i]), col="grey")}

par(new=TRUE) # now overlay plot of weights ~ N

plot(s,r,pch=19,cex=0.75,ylim=c(0,1),xlim=c(0,30),type='b',col='black',ann=F,frame.plot=F)

axis(side=1, at=c(0,50,10,20,30,40))

axis(side=2, at=c(0,1,0.2,0.4,0.6,0.8))

v <- rep(0,length(s))

for (j in 1:length(s)) {v[j] <- var(out3[j,])}

v <- 1.96*sqrt(v/Iterations)

for (i in 1:length(s)){

lines(c(s[i],s[i]),c(r[i]-v[i],r[i]+v[i]))}

abline(v=c(seq(0,50,5)), col='lightgray', lty=3)

abline(h=c(seq(0,1,0.1)), col='lightgray', lty=3)

dev.off()

# end of code
