# Supplementary material for: A solution to minimum sample size for regressions
Source: PLoS One. 2020 Feb 21;15(2):e0229345. doi: 10.1371/journal.pone.0229345 (PMC7034864; doi:10.1371/journal.pone.0229345)
Supplement: S1 Table — Variance was set as standard deviation (σ) of residuals. Coefficients (α, β, γ) designate data shape, where α is the intercept, β is a multiplier for x (as in y = α + β x), and γ is a multiplier for the quadratic term (… + γ x2). (DOCX) [file pone.0229345.s003.docx]

**S2 Table. Coefficients and variance used to make data sets.** Variance was set as standard deviation (*σ*) of residuals. Coefficients (*α*, *β*, *γ*) designate data shape, where *α* is the intercept, *β* is a multiplier for x (as in y = *α* + *β* x), and *γ* is a multiplier for the quadratic term (... + *γ* x^2^).

| Data shape | *σ* | *α* | *β* | *γ* |
| --- | --- | --- | --- | --- |
| Null – high *σ* | 20 | 50 | - - | - - |
| Null – low *σ* | 5 | 50 | - - | - - |
| Straight-line – high *σ*, low slope | 10 | 1 | 2 | - - |
| Straight-line – high *σ*, high slope | 10 | 1 | 2 | - - |
| Straight-line – low *σ*, low slope | 1 | 1 | 0.25 | - - |
| Straight-line – low *σ*, low slope | 1 | 1 | 0.25 | - - |
| Quadratic – high *σ*, low *β*, low *γ* | 5 | 1 | 3 | -0.015 |
| Quadratic – high *σ*, low *β*, high *γ* | 4.5 | 1 | 5 | -0.1 |
| Quadratic – high *σ*, high *β*, low *γ* | 10 | 1 | 3 | -0.015 |
| Quadratic – high *σ*, high *β*, high *γ* | 50 | 1 | 5 | -0.1 |
| Quadratic – low *σ*, low *β*, low *γ* | 1 | 1 | 1 | -0.01 |
| Quadratic – low *σ*, low *β*, high *γ* | 1 | 1 | 1 | -0.025 |
| Quadratic – low *σ*, high *β*, low *γ* | 1 | 1 | 3 | -0.025 |
| Quadratic – low *σ*, high *β*, high *γ* | 1 | 1 | 5 | -0.1 |
